# Relationship between initial B-type natriuretic peptide levels and detection of atrial fibrillation with an insertable cardiac monitor in cryptogenic stroke: CRYPTON-ICM registry

**DOI:** 10.3389/fneur.2024.1436062

**Published:** 2024-09-18

**Authors:** Takuya Moriyama, Kenichi Todo, Hiroshi Yamagami, Yoko Kimura, Shiro Yamamoto, Keiko Nagano, Ryosuke Doijiri, Hidekazu Yamazaki, Kazutaka Sonoda, Junpei Koge, Taira Nakayama, Tomonori Iwata, Yuji Ueno, Yasufumi Gon, Shuhei Okazaki, Tsutomu Sasaki, Hideki Mochizuki

**Affiliations:** ^1^Department of Neurology, Osaka University Graduate School of Medicine, Suita, Japan; ^2^Department of Neurology, NHO Osaka National Hospital, Osaka, Japan; ^3^Division of Stroke Prevention and Treatment, Institute of Medicine, University of Tsukuba, Tsukuba, Japan; ^4^Department of Neurology, Iwate Prefectural Central Hospital, Morioka, Japan; ^5^Department of Neurology, Yokohama Shintoshi Neurosurgical Hospital, Yokohama, Japan; ^6^Department of Neurology, Saiseikai Fukuoka General Hospital, Fukuoka, Japan; ^7^Department of Cerebrovascular Medicine, National Cerebral and Cardiovascular Center, Osaka, Japan; ^8^Department of Neurology, Tokai University, Isehara, Japan; ^9^Department of Neurology, Juntendo University Faculty of Medicine, Bunkyo, Japan

**Keywords:** atrial fibrillation, B-type natriuretic peptide, cryptogenic stroke, insertable cardiac monitor, CRYPTON-ICM registry

## Abstract

High B-type natriuretic peptide (BNP) levels are associated with new atrial fibrillation (AF). This study investigated the distribution of AF detection rates according to BNP levels in patients with cryptogenic stroke (CS) using an insertable cardiac monitor (ICM). We enrolled consecutive patients with CS who underwent ICM implantation between October 2016 and September 2020 at eight stroke centers in Japan. Those with BNP levels were divided into three groups by tertiles. We evaluated the association of BNP levels with AF detection. Youden’s index was calculated to identify the optimal cutoff for BNP. Of 417 patients, we analyzed 266 patients with BNP data. The tertile range of BNP level was 19.0 to 48.5 pg/mL. AF detection rate was 13.3%/year, 12.8%/year, and 53.7%/year in the low-BNP (≤19.0), mid-BNP (19.1–48.4), and high-BNP (≥48.5) groups, respectively (log-rank trend *p* < 0.01). Compared with low-BNP group, the adjusted hazard ratios for AF detection in mid-and high-BNP groups were 0.91 [95% confidence interval (CI) 0.46–1.78] and 2.17 (95% CI 1.14–4.13), respectively. Receiver operating characteristic curve analysis showed the optimal cutoff value was 43.4 pg/mL. The area under curve using BNP to predict AF detection was 0.69. The BNP level was associated with AF detection in patients with CS. This relationship changed around the BNP levels of 40–50 pg/mL.

## Introduction

Cryptogenic stroke (CS) is a stroke with an undetermined cause, even after sufficient diagnostic workup (1), and accounts for 9–25% of patients with ischemic stroke (2). Covert atrial fibrillation (AF) is one of the major causes of CS. Moreover, previous trials using direct oral anticoagulants for patients with CS failed to reduce the risk of recurrent stroke compared to antiplatelet agents (3, 4). Therefore, the detection of AF episodes in patients with CS and the use of appropriate anticoagulants are crucial for the prevention of recurrent strokes.

In the past few years, non-invasive long-term electrocardiogram (ECG) or insertable cardiac monitoring (ICM) has been widely used to detect AF in patients with CS. The 30 Day Event Monitoring Belt for Recording Atrial Fibrillation After a Cerebral Ischemic Event (EMBRACE) study (5) and the Cryptogenic Stroke and Underlying AF (CRYSTAL AF) trial (6) showed a higher rate of AF detection in patients with long-term monitoring than in those with regular monitoring. Therefore, the use of these devices and prolonged rhythm monitoring for approximately 30 days in patients with CS is recommended in the guidelines for the secondary prevention of stroke (7).

Previous studies have shown that several factors such as age, high B-type natriuretic peptide (BNP) or N-terminal pro-BNP, dilation of the left atrial diameter (LAD), frequent premature atrial contractions (PAC), large vessel occlusion (LVO), and low left ventricle ejection fraction (LVEF) were associated with AF detection in patients with CS (8–14). In a systematic review and meta-analysis, extended duration of ICM and increased patient age were strongly associated with increased AF detection (15).

Although it has been suggested that elevated BNP levels are associated with an increased AF detection rate proposing a cutoff BNP level associated with new AF (16–20) in patients with CS, the distribution of AF detection rates according to BNP levels is uncertain. Therefore, the present study aimed to investigate the distribution of AF detection rates according to BNP levels and identify the threshold BNP level that leads to more efficient AF detection using ICM in patients with CS.

## Materials and methods

### Ethical approval

This study was conducted in accordance with the Ethical Guidelines for Medical and Health Research Involving Human Subjects in Japan and the Declaration of Helsinki guidelines for investigations involving human subjects, and all methods were performed in accordance with the relevant guidelines and regulations for observational studies. This study was approved by the institutional review boards of all eight centers. Because of the retrospective nature of this study, written informed consent was not required.

### Patients

The CRYPTON-ICM (CRYPTOgenic stroke evaluation in Nippon using Insertable Cardiac Monitor) registry is a retrospective observational study that enrolled consecutive patients with CS who underwent ICM implantation between October 2016 and September 2020 at eight stroke centers in Japan. It was registered at http://www.umin.ac.jp/ctr/ (UMIN000044366), and the participating stroke centers were as follows: Iwate Prefectural Central Hospital, National Cerebral and Cardiovascular Center, Yokohama Shintoshi Neurosurgical Hospital, Saiseikai Fukuoka General Hospital, National Hospital Organization Osaka National Hospital, Juntendo University, Tokai University, and Osaka University. The institutional review board of each institute approved this study.

The inclusion criteria for this registry were as follows: (1) patients diagnosed with CS based on the Trial of Org 10,172 in Acute Stroke Treatment (TOAST) criteria (21) and (2) patients who underwent ICM implantation for AF detection. This study of the CRYPTON-ICM registry included patients whose BNP levels were measured within 30 days after the onset of index stroke. Indications for ICM implantation were determined according to the Japanese clinical guidelines for ICM in patients with CS (1): magnetic resonance imaging, transthoracic echocardiography, 12-lead ECG, ambulatory ECG monitoring for 24 h or more, and imaging of both the extracranial and intracranial arteries supplying the area of brain ischemia (catheter, magnetic resonance, computed tomography angiography, or cervical duplex ultrasonography) are strongly recommended. Additionally, transesophageal echocardiography, ultrasonic examination for right-to-left shunt, venous duplex ultrasonography, blood tests on thrombosis-hemostasis, and other parameters for stroke are also recommended. Based on these examinations, we underwent ICM implantation for the patients with non-lacunar ischemic stroke, absence of extracranial or intracranial stenosis ≥50% in arteries supplying the ischemic area, no major risk cardioembolic source, and aortic plaques by transthoracic or transesophageal echocardiography and cardiac monitoring for 24 h or more, and no other specific cause of stroke identified, including arteritis, dissection, migraine, vasospasm, drug abuse, and thrombophilia.

Patients were divided into three groups according to the tertile ranges of BNP levels, into low-, mid-, and high-BNP groups.

### ICM implantation and AF detection

ICMs [Reveal LINQ (Medtronic, Minneapolis, MN, United States), Confirm Rx (Abbott Laboratories, Lake Bluff, IL, United States) or BioMonitor 2-AF (Biotronik SE & Co. KG, Berlin, Germany)] were implanted under local anesthesia in the left parasternal position above the fourth intercostal space. The attending physician determined the device selection. All the devices were programmed to detect AF using a unique algorithm that identifies AF by assessing the irregularity of R-R peak intervals. We defined AF as an irregular heart rate lasting ≥2 min. Device data were transmitted to the companies’ server remotely, automatically alerting the study physician when an AF episode was detected, which was judged by the study physician using a remote monitoring system. The most recent ICM data were obtained before March 2021.

### Clinical variables

We obtained the following clinical information from the medical records: age at the time of ICM implantation; sex; hypertension; diabetes mellitus; history of congestive heart failure (CHF); CHADS_2_ score (22) after index stroke; LAD and LVEF based on transthoracic echocardiography; plasma BNP levels; PR interval on ECG; the number of PACs on 24-h Holter ECG monitoring; LVO at the index stroke; and number of days from the index stroke onset to BNP level measurement, ICM implantation, and the first AF episode. Data before ICM implantation were used; if not available, data after ICM implantation and before AF detection were used. We stratified the continuous variables according to clinically relevant thresholds. Frequent PACs were defined as >200/day (13). LVO was defined as occlusion of the internal carotid artery, middle cerebral artery (M1), or basilar artery.

### Study outcome

The primary outcome was AF detection with ICM. The difference in the AF detection rate among the groups varying in BNP levels was analyzed. The optimal cutoff point of BNP levels to discriminate AF detection was also evaluated.

### Statistical analysis

Patients were divided into three groups according to the tertile ranges of BNP levels, into low-, mid-, and high-BNP groups. Continuous variables are presented as mean ± SD or median values and interquartile ranges (IQRs) and analyzed using ANOVA or Kruskal–Wallis test among the groups varying in BNP levels. Categorical variables are presented as numbers and percentages and analyzed using the chi-square test. Survival analysis was performed using Kaplan–Meier curves followed by the log-rank test. The time until the first incidence of AF was analyzed as a time-to-event variable using the calendar date on which AF rhythm was observed on ICM or censoring on the final day of ICM monitoring if AF was never observed. Cox proportional hazards models were developed to analyze the association of BNP levels with AF detection. Multivariate stepwise Cox regression models were developed adjusting for parameters associated with each outcome in the univariate analysis (*p* < 0.05). Additionally, Youden’s index was calculated to identify the optimal BNP level cutoff points to determine AF detection using receiver operating characteristic (ROC) curves. To investigate the distribution of AF detection rate in more detail, the patients were divided by the cutoff value, and each group was further divided by the median value, resulting in 4 groups. Multivariate stepwise logistic regression models were developed adjusting for parameters associated with each outcome in the univariate analysis (*p* < 0.05). Statistical significance was set at *p* < 0.05. All analyses were conducted using JMP (version 15.1.0; SAS Institute, Cary, NC, United States).

## Results

### Baseline characteristics

Among the 417 patients enrolled in the CRYPTON-ICM registry, 101 patients without BNP data and 50 patients measured beyond 30 days after the onset of index stroke were excluded. Thus, 266 patients [mean age, 68.1 ± 12.0 years; male, *n* = 167 (62.8%)] were included in this study. The median number of days from index stroke to BNP measurement was 1 day (IQR, 0–3 days), and to ICM implantation day was 20 days (IQR, 12–47 days). The median follow-up period for ICM was 579 days (IQR, 201–807 days).

**Figure 1:**
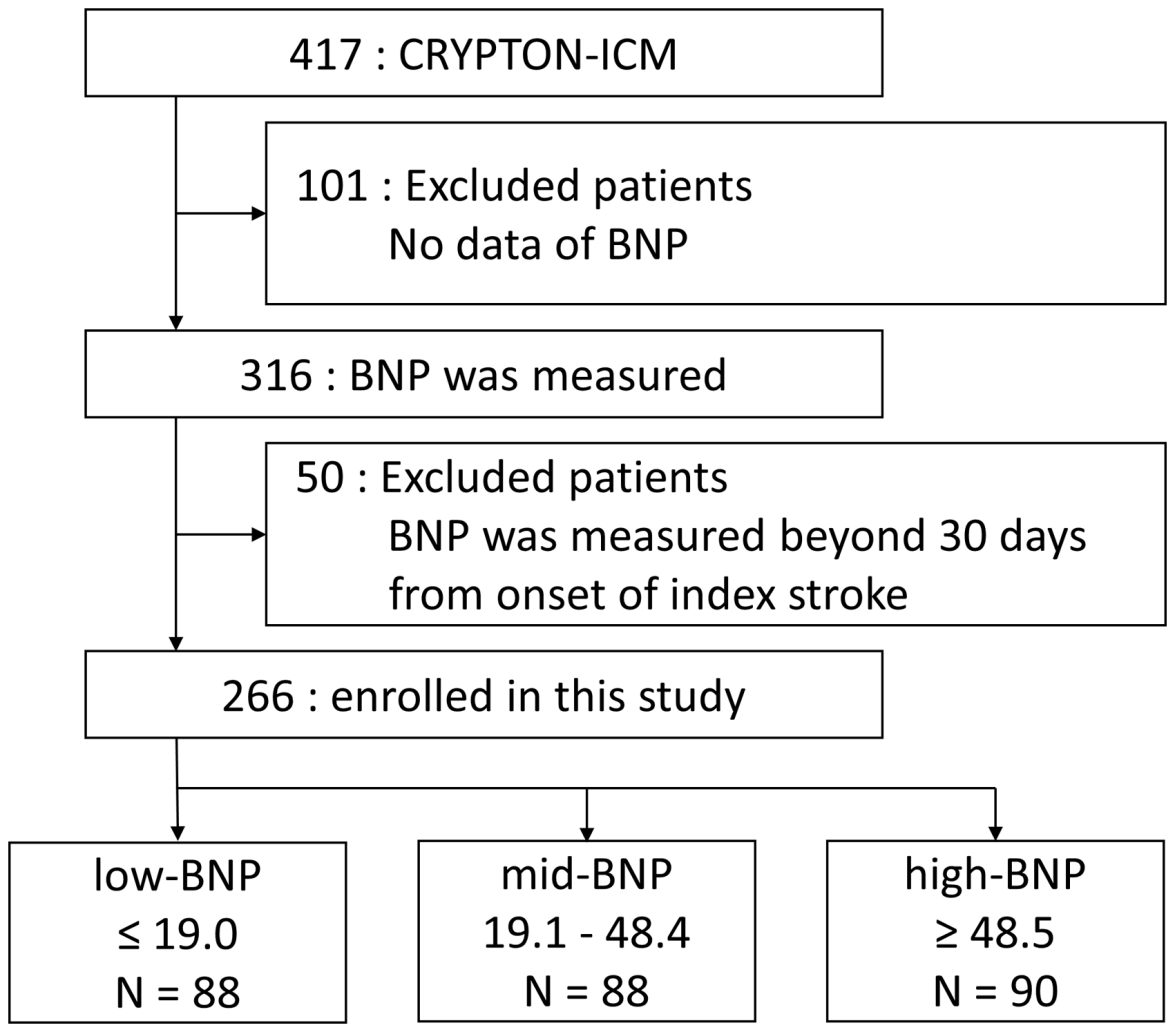
Flowchart for exclusion criteria used in the study and the division of BNP groups. Patients whose BNP levels were measured within 30 days of index stroke onset were included in this study. BNP, B-type natriuretic peptide.

Patients were divided according to the tertile ranges of BNP levels into low-BNP (≤19.0 pg/mL), mid-BNP (19.1–48.4 pg/mL), and high-BNP (≥48.5 pg/mL) groups. A flowchart of the exclusion criteria of the patients is illustrated in Figure 1. Patient clinical characteristics are summarized in Table 1. The patients in the high-BNP group were older than those in the other groups. Histories of hypertension, diabetes mellitus, and CHF did not differ among the three BNP-level groups. Frequent PACs were higher in the high-BNP group than in the other groups (9% for the low-BNP group vs. 21% for the mid-BNP group vs. 40% for the high-BNP group; *p* < 0.01). Patients in the high-BNP group had a larger LAD than the other groups (mean 33.5 ± 4.35 mm vs. 34.9 ± 5.5 mm vs. 36.2 ± 6.54 mm, *p* = 0.01). No significant differences were noted in LVEF among the three groups. Comparing with 151 patients without BNP data within 30 days after stroke onset, these 266 patients had a lower rate of hypertension, a smaller LAD, and a shorter time from stroke onset to ICM implantation, but a similar AF detection rate (Supplementary Table S1).

**Table 1:** Baseline characteristics.

	Low-BNP (≤19.0) *n* = 88	Mid-BNP (19.1–48.4) *n* = 88	High-BNP (≥48.5) *n* = 90	*p*-value^*^
Age, years, mean ± SD	62.6 ± 11.3	67.2 ± 11.5	74.2 ± 10.2	<0.01
Male, *n* (%)	59 (67.1)	55 (62.5)	53 (58.9)	0.53
Hypertension, *n* (%)	46 (52.3)	43 (48.9)	59 (65.6)	0.060
Diabetes mellitus, *n* (%)	20 (22.7)	14 (15.9)	16 (17.8)	0.49
History of CHF, *n* (%)	3 (3.41)	3 (3.41)	5 (5.56)	0.71
CHADS_2_ score, median (IQR)	3 (2–4)	3 (2–3)	3 (3–4)	0.15
1st degree atrioventricular block, *n* (%) (*n* = 264)	11 (12.5)	14 (16.0)	23 (25.6)	0.059
PAC ≥200/day, *n* (%) (*n* = 243)	9 (11.1)	21 (26.3)	40 (48.8)	<0.01
LAD, mm, mean ± SD (*n* = 264)	33.5 ± 4.35	34.9 ± 5.50	36.2 ± 6.54	0.01
LVEF, %, mean ± SD (*n* = 264)	65.3 ± 5.57	66.1 ± 6.59	65.6 ± 7.24	0.75
Large vessel occlusion, *n* (%)	13 (14.8)	16 (18.2)	18 (20.0)	0.65
Index stroke to BNP measure, days, median (IQR)	1 (0–3)	1 (0–3)	1 (0–2)	0.53
BNP measure to ICM implantation, days, median (IQR)	23 (10–65)	16 (9–31)	17 (9–48)	0.18
Index stroke to ICM implantation, days, median (IQR)	28 (13–66)	19 (12–36)	19 (12–49)	0.09
Devices				0.78
Reveal LINQ (%)	79 (89.8)	80 (90.9)	85 (94.5)	
Confirm Rx (%)	7 (8.0)	7 (8.0)	4 (4.4)	
BioMonitor 2-AF (%)	2 (2.2)	1 (1.1)	1 (1.1)	

^*^ANOVA or Kruskal–Wallis test and chi-square tests were used for comparisons. BNP, B-type natriuretic peptide; CHF, congestive heart failure; ICM, insertable cardiac monitor; IQR, interquartile range; LAD, left atrial diameter; LVEF, left ventricular ejection fraction; SD, standard deviation PAC, premature atrial contraction.

### BNP levels and AF detection

**Figure 2:**
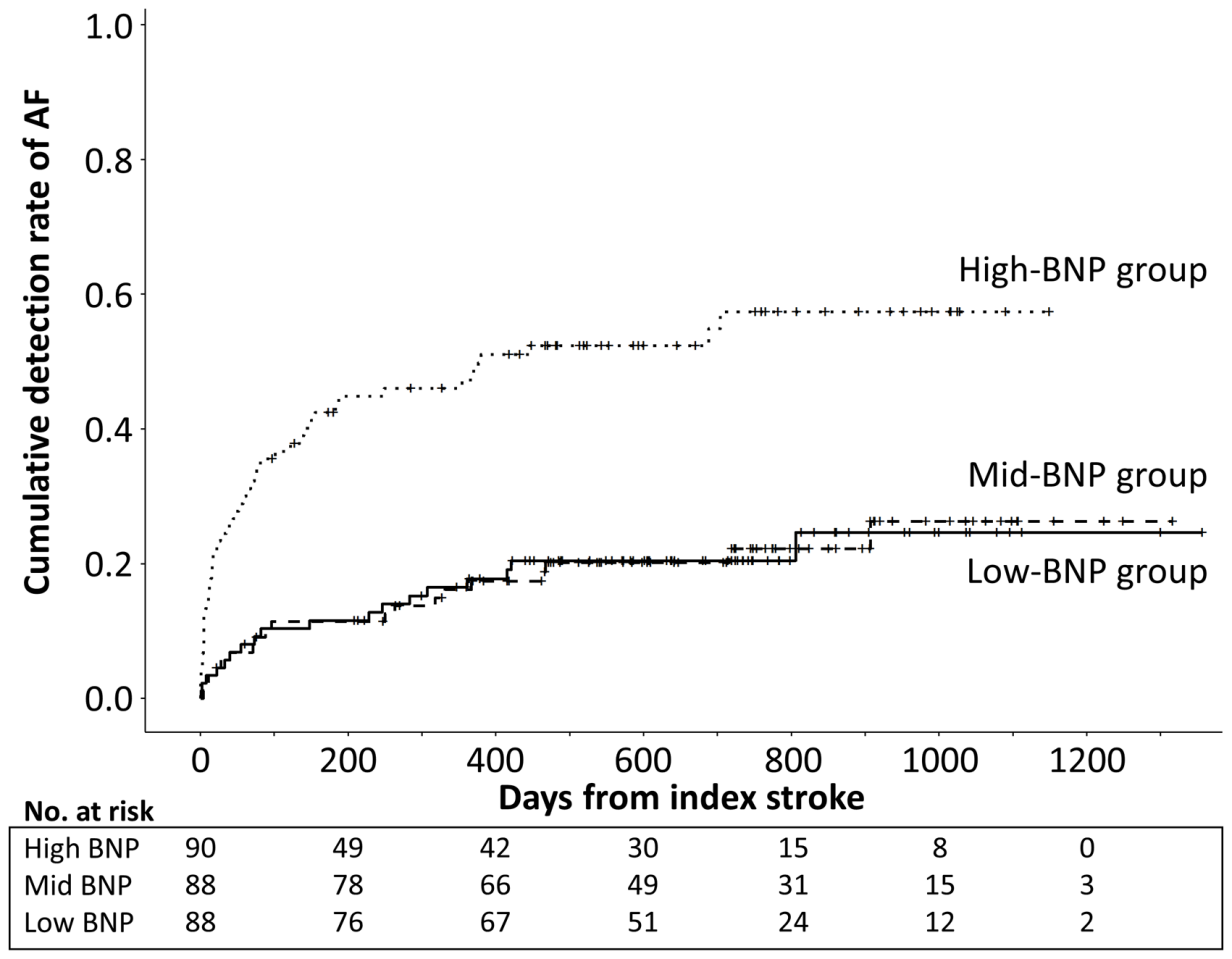
Kaplan–Meier curve estimates from the onset of index stroke to AF detection according to BNP tertile levels. Cumulative AF detection rate was significantly higher in high-BNP group than low-BNP and mid-BNP groups (log-rank *p* < 0.01). AF, atrial fibrillation; BNP, B-type natriuretic peptide.

Of the 266 patients, 85 (32.0%) had ICM-reported AF during the follow-up period. The median time from the index stroke to the first AF episode was 111 days (IQR, 46–275 days). Figure 2 illustrates the Kaplan–Meier curves estimated from the onset of index stroke to AF detection. AF detection rate was 13.3%/year, 12.8%/year, and 53.7%/year in the low-, mid-, and high-BNP groups, respectively (log-rank trend *p* < 0.01). Compared to the low-BNP group, the adjusted hazard ratios for AF detection in the mid-BNP and high-BNP groups were 0.91 [95% confidence interval (CI) 0.46–1.78, *p* = 0.77] and 2.17 (95% CI 1.14–4.13, *p* = 0.02), respectively (Table 2).

**Table 2:** Univariate and multivariate Cox regression models for AF detection.

Variables	Crude HR	95% CI	*p*-value	Adjusted HR^*^	95% CI	*p*-value
Age (/1 Y)	1.03	1.01–1.06	<0.01	1.01	0.99–1.03	0.48
Male	0.91	0.59–1.40	0.66	—	—	—
Hypertension	1.18	0.77–1.82	0.45	—	—	—
Diabetes mellitus	1.09	0.64–1.85	0.76	—	—	—
History of CHF	0.85	0.27–2.70	0.79	—	—	—
CHADS_2_ score (/1 point)	1.17	0.95–1.43	0.13	—	—	—
1st degree atrioventricular block (*n* = 264)	1.05	0.61–1.81	0.86	—	—	—
PAC ≥200/day (*n* = 243)	2.82	1.81–4.40	<0.01	2.04	1.23–3.38	<0.01
LAD (/1 mm) (*n* = 264)	1.04	1.01–1.08	0.022	1.02	0.98–1.06	0.35
LVEF (/1%) (*n* = 264)	1.00	0.97–1.03	0.99	—	—	—
Large vessel occlusion	1.30	0.77–2.18	0.33	—	—	—
Index stroke to BNP measure (/1 day)	1.00	0.97–1.04	0.85	—	—	—
BNP measure to ICM implantation (/1 day)	1.01	0.99–1.00	0.086	—	—	—
Index stroke to ICM implantation (/1 day)	1.00	0.99–1.00	0.088	—	—	—
BNP tertile						
Low-BNP (≤19.0)	1 (reference)	—	—	1 (reference)	—	—
Mid-BNP (19.1–48.4)	1.01	0.53–1.92	0.98	0.91	0.46–1.78	0.77
High-BNP (≥48.5)	3.43	1.99–5.90	<0.01	2.17	1.14–4.13	0.019

^*^Multivariate Cox regression model was developed adjusting for the variables associated with AF detection in the univariate analysis (*n* = 241). BNP, B-type natriuretic peptide; CHF, chronic heart failure; CI, confidence interval; HR, hazard ratio; ICM, insertable cardiac monitor; LAD, left atrial diameter; LVEF, left ventricular ejection fraction; PAC, premature atrial contraction.

**Figure 3:**
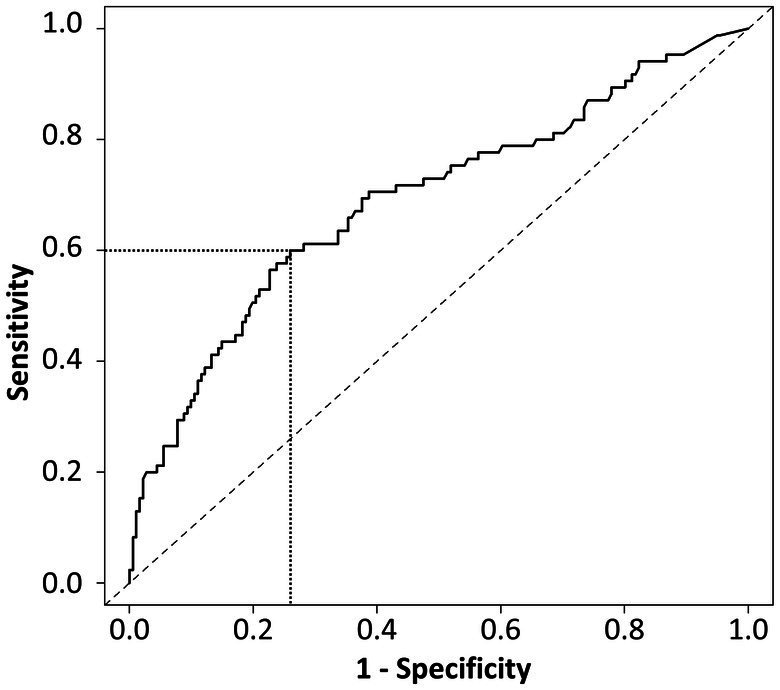
Receiver operating characteristic curve analysis. The optimal cutoff level of BNP, and sensitivity and specificity required to discriminate AF detection was BNP 43.4 pg/mL, 60.0 and 74.0%, respectively. The area under curve using BNP to predict AF detection was 0.69. AF, atrial fibrillation; BNP, B-type natriuretic peptide.

In addition, ROC analysis was performed. The optimal BNP cutoff level to discriminate AF detection was 43.4 pg/mL, corresponding with a sensitivity of 60.0% and a specificity of 74.0%. The area under the curve using BNP to predict AF detection was 0.69 (Figure 3). Patients were divided by this cutoff level, 43.4 pg/mL. The median values in patients with BNP levels below and above 43.4 pg/mL were 18.1 pg/mL and 100.0 pg/mL, respectively, and patients were divided into 4 groups based on these values. AF detection rates were 13.9%/year, 11.6%/year, 39.9%/year, and 65.1%/year in patients with BNP levels ≤18.1, >18.1 to ≤43.4, >43.4 to ≤100.0, and >100 pg/mL, respectively (log-rank trend *p* < 0.01) (Supplementary Figure S1). Compared to patients with BNP of ≤18.1, the adjusted hazard ratios for AF detection in those with BNP of >18.1 to ≤43.4, >43.4 to ≤100.0, and >100 pg/mL were 0.83 (95% CI 0.41–1.66; *p* = 0.59), 1.62 (95% CI 0.78–3.35, *p* = 0.19) and 2.47 (95% CI 1.24–4.93, *p* = 0.010), respectively (Supplementary Table S2).

### Comparison between devices

The percentage of devices implanted was comparable in each group (Table 1). The AF detection rate was not different between devices (Supplementary Table S3).

## Discussion

This study showed that elevated BNP levels in the acute phase of CS are associated with an increased AF detection rate with ICM. The AF detection rate among the BNP-level groups was higher in the high-BNP group (≥48.5 pg/mL) than in the low-and mid-BNP groups. Although the AUC values were low, an almost equivalent threshold level was also obtained in ROC analysis (43.4 pg/mL). This relationship between BNP and AF detection changed around the BNP levels of 40–50 pg/mL.

Previous studies have shown that high BNP levels are associated with AF detection using 65–131 pg/mL thresholds, based on Youden’s index, and the highest tertile, or the highest quartile (16–20). The discrepancy in BNP thresholds between previous and current studies may be partly due to differences in study design. Previous studies included patients with acute ischemic stroke without AF on admission (16, 17, 20). Additionally, participants in one of the previous studies were patients with acute non-lacunar stroke without AF after 3 days of conventional screening (19). In contrast, our study included patients with ischemic stroke without AF after 20 days of conventional screening. Therefore, cases in which AF was detected within a few days of stroke onset were excluded, and the higher BNP levels in these excluded cases may have led to a lower BNP threshold than that previously reported. Furthermore, the longer monitoring period in our study may be related to a lower BNP threshold level. In previous studies, the duration of ECG monitoring with non-invasive devices was 1–28 days, and the AF detection rate was 6.8–22.9% (16, 17, 19, 20). According to a systematic review and meta-analysis, a more extended monitoring period is related to a higher AF detection rate (15). In our study, the AF detection rate was as high as 53.3% in the high-BNP group, which could be attributed to the long median monitoring period of 579 days. Additionally, long-term ECG monitoring with ICM increased the sensitivity of AF detection and possibly resulted in the low BNP threshold level.

Nonetheless, this study had some limitations. First, we did not collect information on anemia and renal function, which may affect BNP levels (23, 24). Second, this was a retrospective study, which may have led to potential selection bias. Third, one-third of the patients registered with CRYPTOM-ICM were excluded from this analysis, which may have created an additional selection bias. Although some background characteristics differed between patients included and excluded from this analysis, the frequency of AF detection was similar between groups. Forth, the algorithms of each device to detect AF were partially published and not comparable. The differences in the devices used in this study may have affected the results. However, as there was no difference in AF detection rates between devices and no difference in the percentage of devices used in the three BNP groups, the association between BNP and AF detection was evaluated in this study by integrating patients who used the three devices. Fifth, because this study was a retrospective observational study, the clinical variables were collected from routine inpatients testing or monitoring without specific protocol. Prospective studies with large sample sizes are required to validate the relationship between the timing of BNP measurement and subsequent AF detection. Sixth, some analyses were contrary to known reports. Previous reports have shown that age is strongly associated with AF (15), but the present study found no association after adjustment. This does not rule out an association between age and AF and is considered a type II error due to the small number of cases. Furthermore, an increase in age is correlated with an increase in BNP (25). Additionally, our study results demonstrated a correlation coefficient between BNP and age [*r* = 0.36 (*p* < 0.01)]. This may explain why age does not emerge as a predictor.

## Conclusion

In conclusion, the BNP level was associated with AF detection in patients with cryptogenic stroke. This relationship changed around the BNP levels of 40–50 pg/mL. These findings may be helpful in the decision of ICM implantation in individual patients with cryptogenic stroke.

## Data Availability

The raw data supporting the conclusions of this article will be made available by the authors, without undue reservation.
