# Supplementary material for: Relationship between initial B-type natriuretic peptide levels and detection of atrial fibrillation with an insertable cardiac monitor in cryptogenic stroke: CRYPTON-ICM registry
Source: Front Neurol. 2024 Sep 18;15:1436062. doi: 10.3389/fneur.2024.1436062 (PMC11445933; doi:10.3389/fneur.2024.1436062)
Supplement: Supplementary file 1 [file Data_Sheet_1.docx]

**Supplemental Table 1: Characteristics of the patients included and excluded from this study**

|  | Included  (N = 266) | Excluded  (N = 151) | *P* value |
| --- | --- | --- | --- |
| Age, years, mean ± SD | 68.1 ± 12.0 | 67.4 ± 12.1 | 0.60 |
| Male, n (%) | 167 (62.8) | 103 (68.2) | 0.26 |
| Hypertension, n (%) | 148 (55.6) | 102 (67.6) | 0.017 |
| Diabetes mellitus, n (%) | 50 (18.8) | 36 (23.8) | 0.22 |
| History of CHF, n (%) | 11 (4.14) | 3 (1.99) | 0.24 |
| CHADS2 score, median (IQR) | 3 (2–4) | 3 (3–4) | 0.24 |
| 1^st^ degree atrioventricular block, n (%) (N = 413) | 48 (18.2) | 20 (13.4) | 0.21 |
| PAC ≥ 200/day, n (%) (N = 389) | 70 (28.8) | 35 (24.0) | 0.30 |
| LAD, mm, mean ± SD (N = 413) | 34.9 ± 5.63 | 37.0 ± 6.02 | < 0.01 |
| LVEF, %, mean ± SD (N = 413) | 65.7 ± 6.49 | 65.9 ± 6.63 | 0.72 |
| Large vessel occlusion, n (%) | 47 (17.7) | 11 (7.3) | < 0.01 |
| Index stroke to BNP measure, days, median (IQR) | – | 1 (0–3) | – |
| BNP measure to ICM implantation, days, median (IQR) | – | 18 (10–44) | – |
| Index stroke to ICM implantation, days, median (IQR) | 20 (12–47) | 23 (14–72) | 0.035 |
| BNP, pg/mL, median (IQR) | 31.2 (14.0–72.3) |  |  |
| BNP > 100, n (%) | 49 (18.4) |  |  |
| NT-proBNP, pg/mL, median (IQR) (N = 75) |  | 122 (57–271) |  |
| NT-proBNP > 300, n (%) (N = 75) |  | 17 (22.7) |  |
| AF detection, n (%) | 85 (32.0) | 42 (29.5) | 0.60 |

Wilcoxon test and chi-square tests were used for comparisons.

BNP, B-type natriuretic peptide; CHF, congestive heart failure; ICM, insertable cardiac monitor; IQR, interquartile range; LAD, left atrial diameter; LVEF, left ventricular ejection fraction; SD, standard deviation PAC, premature atrial contraction.

**Supplemental Figure 1. Kaplan–Meier curve estimates from the onset of index stroke to AF detection in 4 groups divided by cutoff values determined from the ROC curve and the median of each group.**


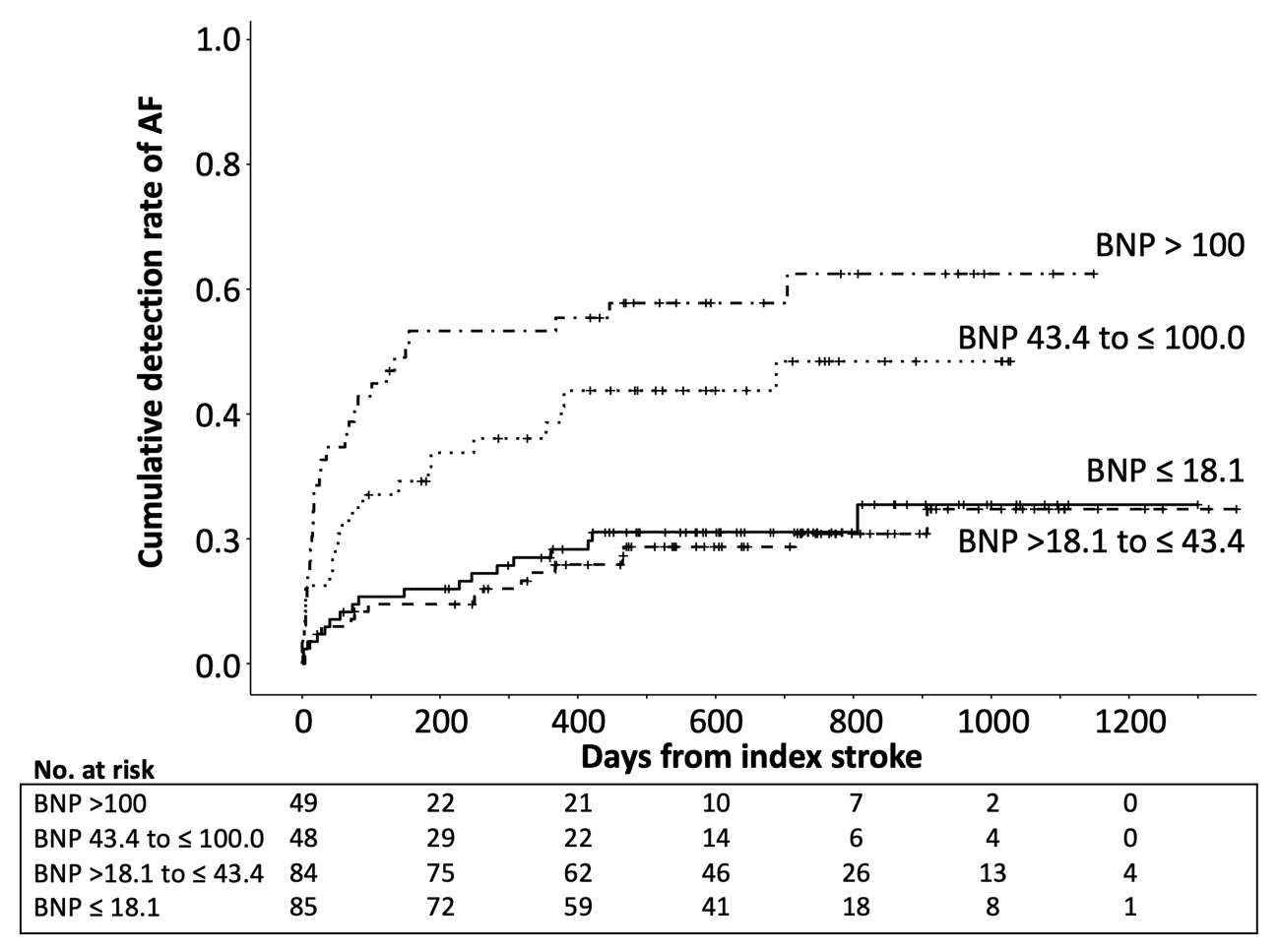


Cumulative AF detection rate was significantly higher in patients with higher BNP levels than lower BNP levels (log-rank P < 0.01).

AF, atrial fibrillation; BNP, B-type natriuretic peptide.

**Supplemental Table 2. Univariate and multivariate Cox regression models for AF detection**

| BNP level (pg/mL) | Crude HR | 95% CI | *P* value | Adjusted HR^*^ | 95% CI | *P* value |
| --- | --- | --- | --- | --- | --- | --- |
| Age (/1 Y) | 1.03 | 1.01–1.06 | <0.01 | 1.01 | 0.98–1.03 | 0.50 |
| PAC ≥ 200/day (n=243) | 2.82 | 1.81–4.40 | 0.01 | 2.12 | 1.28–3.51 | < 0.01 |
| LAD (/1 mm) (n=264) | 1.04 | 1.01–1.08 | 0.02 | 1.01 | 0.98–1.05 | 0.48 |
| BNP level |  |  |  |  |  |  |
| ≤ 18.1 | 1 (reference) | – | – | 1 (reference) | – | – |
| >18.1 to ≤43.4 | 0.90 | 0.46–1.74 | 0.75 | 0.83 | 0.41–1.66 | 0.59 |
| > 43.4 to ≤ 100.0 | 2.49 | 1.33–4.68 | < 0.01 | 1.62 | 0.78–3.35 | 0.19 |
| > 100 | 3.91 | 2.17–7.06 | < 0.01 | 2.47 | 1.24–4.93 | 0.010 |

*Multivariate Cox regression model was developed adjusting for the variables associated with AF detection in the univariate analysis (n=241).

BNP, B-type natriuretic peptide; CI, confidence interval; HR, hazard ratio; ICM, insertable cardiac monitor; LAD, left atrial diameter; PAC, premature atrial contraction.

**Supplemental Table 3. The AF detection rate with each device.**

|  | Reveal LINQ | Confirm Rx | BioMonitor 2-AF | *P* value |
| --- | --- | --- | --- | --- |
| AF detection rate | 31.6 %/year | 38.9 %/year | 25 %/year | 0.79 |

**Supplemental Table 4. The collaborators for their assistance of data collection.**

| Shintaro Sugiyama, MD | Department of Neurology, Osaka University Graduate School of Medicine |
| --- | --- |
| Mariko Ohara, MD | Department of Neurology, Osaka University Graduate School of Medicine |
| Takeshi Ikegami, MD | Department of Neurology, Osaka University Graduate School of Medicine |
| Tomoya Wadayama, MD | Department of Neurology, Osaka University Graduate School of Medicine |
| Yasutaka Murakami, MD | Department of Neurology, Osaka University Graduate School of Medicine |
| Rei Sakurai, MD | Department of Neurology, Osaka University Graduate School of Medicine |
| Naoto Kimura, MD | Department of Neurosurgery, Iwate Prefectural Central Hospital |
| Masafumi Morimoto, MD | Department of Neurosurgery, Yokohama Shintoshi Neurosurgical Hospital |
| Daisuke Kondo, MD | Department of Neurology, Saiseikai Fukuoka General Hospital |
| Kiyotaka Oi, MD | Department of Neurology, Iwate Prefectural Central Hospital |
| Masatoshi Koga, MD | Department of Cerebrovascular Medicine, National Cerebral and Cardiovascular Center |
| Hajime Ikenouchi, MD | Department of Cerebrovascular Medicine, National Cerebral and Cardiovascular Center |
| Eriko Yamaguchi, MD | Department of Cerebrovascular Medicine, National Cerebral and Cardiovascular Center |
| Shuhei Egashira, MD | Department of Cerebrovascular Medicine, National Cerebral and Cardiovascular Center |
| Eiichiro Nagata, MD | Department of Neurology, Tokai University School of Medicine |
| Shunya Takizawa, MD | Department of Neurology, Tokai University School of Medicine |
| Kazunari Honma, MD | Department of Neurology, Tokai University School of Medicine |
| Taira Nakayama, MD | Department of Neurology, Tokai University School of Medicine |
| Ayana Kadokura, MD | Department of Neurology, Tokai University School of Medicine |
| Hideyuki Hanano, MD | Department of Neurology, Tokai University School of Medicine |
| Nobukazu Miyamoto, MD | Department of Neurology Juntendo University Faculty of Medicine |
