# Supplementary figures and images for: Relationship between initial B-type natriuretic peptide levels and detection of atrial fibrillation with an insertable cardiac monitor in cryptogenic stroke: CRYPTON-ICM registry
Source: Front Neurol. 2024 Sep 18;15:1436062. doi: 10.3389/fneur.2024.1436062 (PMC11445933; doi:10.3389/fneur.2024.1436062)

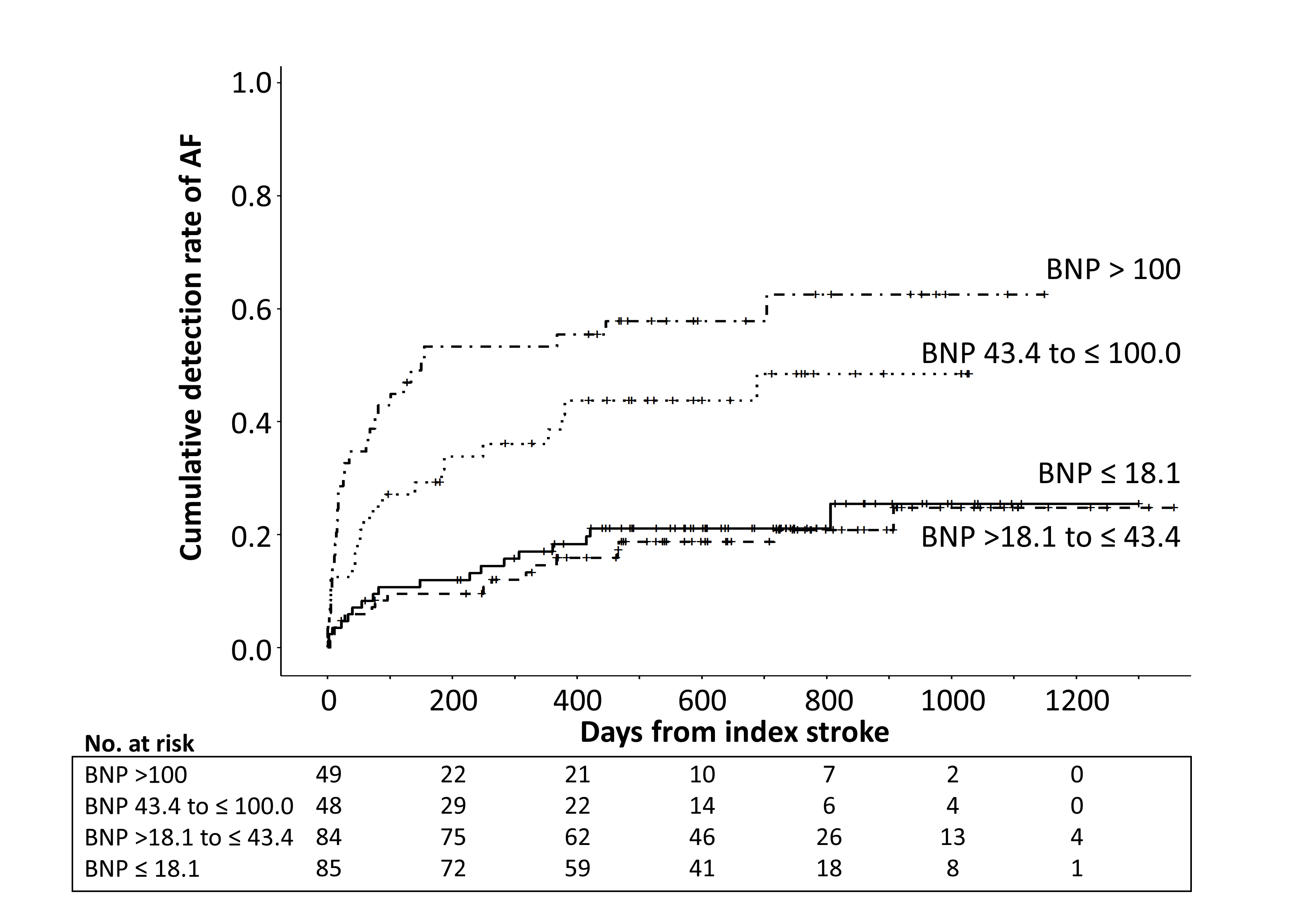

Supplement: Supplementary file 2 [file Image_1.TIF]
